# QTL analysis of novel genomic regions associated with yield and yield related traits in new plant type based recombinant inbred lines of rice (*Oryza sativa* L.)

**DOI:** 10.1186/1471-2229-12-137

**Published:** 2012-08-09

**Authors:** Balram Marathi, Smriti Guleria, Trilochan Mohapatra, Rajender Parsad, Nagarajan Mariappan, Vinod Kunnummal Kurungara, Salwandir Singh Atwal, Kumble Vinod Prabhu, Nagendra Kumar Singh, Ashok Kumar Singh

**Affiliations:** 1 International Rice Research Institute, DAPO Box 7777, Metro Manila, Philippines; 2 Indian Agricultural Research Institute, New Delhi, 110012, India; 3 Central Rice Research Institute, Cuttak, 753006, India; 4 Indian Agricultural Statistical Research Institute, New Delhi, 110012, India; 5 Regional Station, Indian Agricultural Research Institute, Aduthurai, 612101, India; 6 Regional Station, Indian Agricultural Research Institute, Karnal, 132001, India; 7 National Research Centre on Plant Biotechnology, New Delhi, 110012, India

## Abstract

**Background:**

Rice is staple food for more than half of the world’s population including two billion Asians, who obtain 60-70% of their energy intake from rice and its derivatives. To meet the growing demand from human population, rice varieties with higher yield potential and greater yield stability need to be developed. The favourable alleles for yield and yield contributing traits are distributed among two subspecies i.e., *indica* and *japonica* of cultivated rice (*Oryza sativa* L.). Identification of novel favourable alleles in *indica*/*japonica* will pave way to marker-assisted mobilization of these alleles in to a genetic background to break genetic barriers to yield.

**Results:**

A new plant type (NPT) based mapping population of 310 recombinant inbred lines (RILs) was used to map novel genomic regions and QTL hotspots influencing yield and eleven yield component traits. We identified major quantitative trait loci (QTLs) for days to 50% flowering (R^2^ = 25%, LOD = 14.3), panicles per plant (R^2^ = 19%, LOD = 9.74), flag leaf length (R^2^ = 22%, LOD = 3.05), flag leaf width (R^2^ = 53%, LOD = 46.5), spikelets per panicle (R^2^ = 16%, LOD = 13.8), filled grains per panicle (R^2^ = 22%, LOD = 15.3), percent spikelet sterility (R^2^ = 18%, LOD = 14.24), thousand grain weight (R^2^ = 25%, LOD = 12.9) and spikelet setting density (R^2^ = 23%, LOD = 15) expressing over two or more locations by using composite interval mapping. The phenotypic variation (R^2^) ranged from 8 to 53% for eleven QTLs expressing across all three locations. 19 novel QTLs were contributed by the NPT parent, Pusa1266. 15 QTL hotpots on eight chromosomes were identified for the correlated traits. Six epistatic QTLs effecting five traits at two locations were identified. A marker interval (RM3276-RM5709) on chromosome 4 harboring major QTLs for four traits was identified.

**Conclusions:**

The present study reveals that favourable alleles for yield and yield contributing traits were distributed among two subspecies of rice and QTLs were co-localized in different genomic regions. QTL hotspots will be useful for understanding the common genetic control mechanism of the co-localized traits and selection for beneficial allele at these loci will result in a cumulative increase in yield due to the integrative positive effect of various QTLs. The information generated in the present study will be useful to fine map and to identify the genes underlying major robust QTLs and to transfer all favourable QTLs to one genetic background to break genetic barriers to yield for sustained food security.

## Background

Rice is staple food for more than half of the world’s population including two billion Asians, who obtain 60-70% of their energy intake from rice and its derivatives. Rice is globally grown on about 154 million hectares annually with total production of 600 million tons. To meet the growing demand from human population which is expected to touch 9 billion by 2050, in a changing global climatic order, rice varieties with higher yield potential and greater yield stability need to be developed [1]. One of the means of achieving the projected production demand is by integrating classical breeding techniques with modern biotechnological tools for rice improvement [2].

Most of the agronomically important traits are complex and follow quantitative inheritance. Information on the number and chromosomal locations of the genetic loci influencing expression of a trait, their relative contribution to the trait expression, possible pleiotropic effects or epistatic interactions among the loci and their sensitivity to variations in environments are very important for the utilization of these loci for crop improvement. Until 1980s, quantitative traits were studied in terms of population parameters estimated through various mating designs and such analyses were based on number of conditions and assumptions for statistical interpretation to understand genetics and in predicting response to selection. A key development in the field of complex trait analysis was the discovery of DNA based genetic markers, physical establishment of high density genetic maps and development of QTL mapping methodologies such as single marker analysis, interval mapping, composite interval mapping and multi trait mapping [3]. Quantitative traits are influenced by environment and tend to show varied degree of genotype by environment (G × E) interactions [4]. Presence of significant G × E interaction has been reported by comparing QTLs detected in multiple environments. The disappearance of QTLs detected in one environment in another has been considered a manifestation of G × E interaction and the detection of QTLs with consistent expression across environments is considered as stability indicator for the utilization of these QTLs in breeding program [5,6].

There are number of reports of mapping and introgression of QTLs from wild species in rice. However, the related subspecies of *Oryza sativa* such as *japonica* carry many favorable alleles which can be used for improvement of *indica.* It has been observed that derivatives of *indica/japonica* cross have higher yield vigor than either *indica/indica* or *japonica/japonica* derivatives. Therefore identifying the chromosomal locations influencing yield and yield related traits in inter-sub specific derivatives is useful for rice improvement [7]. Exploitation of inter sub specific (*indica* x *japonica*) diversity is conceptualized as new plant type and utilized in pedigree breeding, has been suggested as a possible means for breaking genetic ceiling to yield in rice [8]. The present study is one of the first effort in this direction to use *indica*/*japonica* derivative for identification of QTLs.

The objectives of the present study were to map novel genomic regions and QTL hotspots influencing yield and yield related traits using multi-location phenotyping data from a recombinant inbred population generated by crossing Pusa1266, a new plant type rice genotype derived from *indica* × *japonica* cross with a highly adapted high yielding *indica* rice variety Jaya.

## Results

### Trait performance

**Figure 1:**
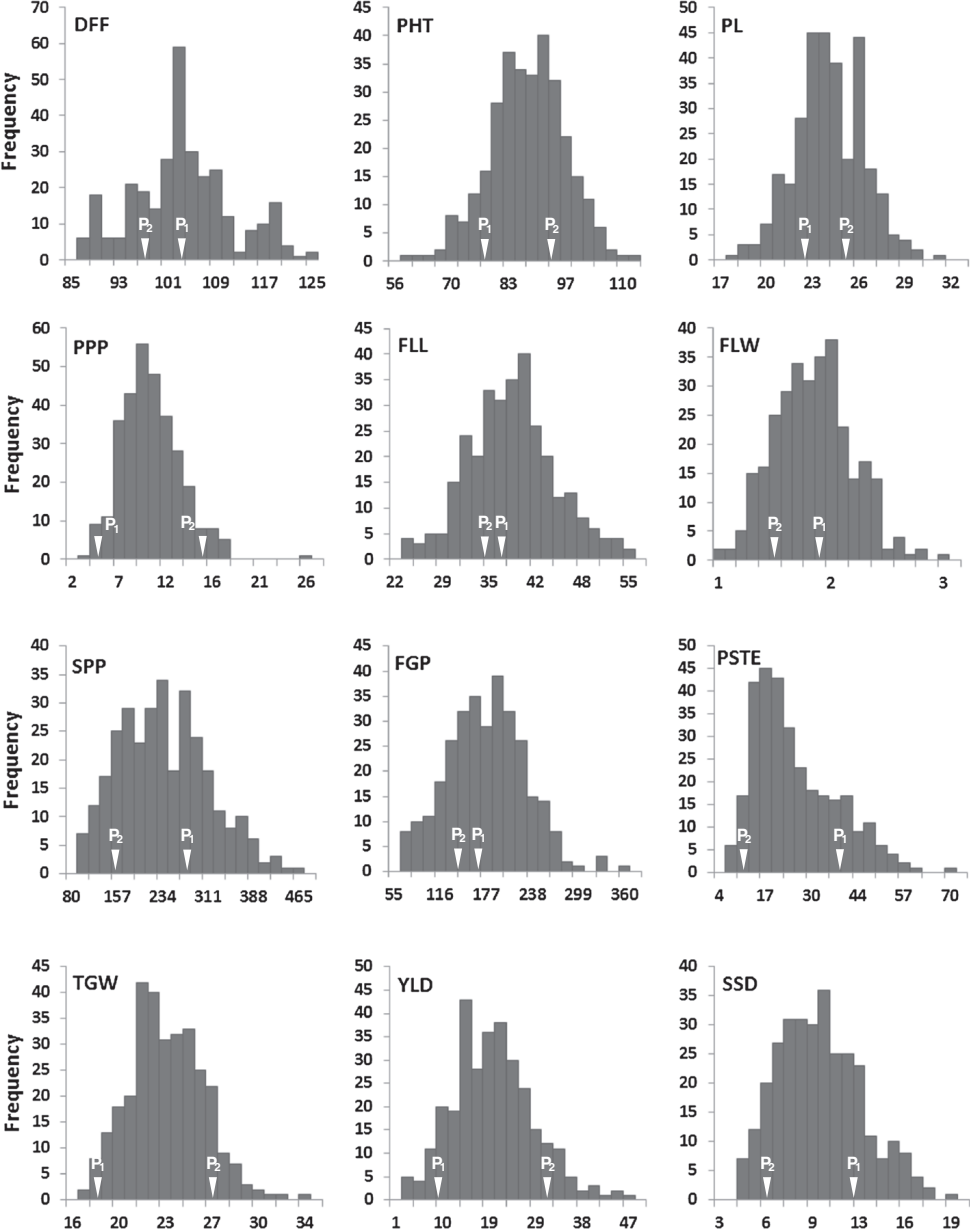
**Frequency distribution of Pusa1266 × Jaya derived recombinant inbred lines for twelve traits at RBGRC, Aduthurai during *Kharif* , 2006 Parental trait means are indicated by arrows.**

The mean performance of parents and minimum and maximum trait values of RILs at three locations are presented in Table 1. Higher phenotypic variation was observed for panicles per plant, grain yield per plant, per cent sterility, spikelets per panicle, filled grains per panicle, spikelet setting density, flag leaf length and flag leaf width. Bi-directional transgressive segregation was observed for all traits. Most of the traits were approximately normally distributed at all three locations (Figure 1). Three traits, i.e. panicle length, panicles per plant and flag leaf width were found to be heterogeneous for error variances hence combined analyses for these traits were performed by transforming data and remaining traits were analyzed using original data. The combined analysis of variance revealed significant differences among RILs for all twelve traits under study (Table 2). Significant effect of location was observed for all traits and block effects were significant for all traits except panicle length and spikelet setting density. Significant interaction between RILs and location was observed for all traits at 1% level of significance except for panicles per plant, which was significant at 5% level of significance.

**Table 1:** **Performance of Pusa1266, Jaya and RILs for yield and yield related traits at New Delhi, Karnal, Aduthurai during *Kharif* , 2006**

**Sl. No**	**Character**	**Location**	**Pusa1266**	**Jaya**	**RILs**				
					**Mean**	**SEd**	**Min**	**Max**	**% transgressive segregants**
1	DFF	New Delhi	103.83	92.77	98.15	1.42	77.00	119.00	55.16
		Karnal	106.52	100.83	104.2	1.65	82	132	51.61
		Aduthurai	103.03	96.63	102.3	2.34	85.0	124.0	29.35
2	PHT	New Delhi	100.27	110.64	104.26	2.79	81.86	137.68	56.77
		Karnal	90.95	100.97	94.92	3.01	74.8	124.4	48.39
		Aduthurai	77.15	93.36	85.90	3.29	55.92	112.90	33.87
3	PL	New Delhi	25.09	28.43	26.05	0.69	19.90	32.94	44.83
		Karnal	22.2	26.49	24.6	0.53	20.32	29.92	26.13
		Aduthurai	22.53	25.37	23.73	1.28	16.48	31.42	49.68
4	PPP	New Delhi	8.46	12.60	10.28	1.60	5.40	19.40	42.90
		Karnal	7.37	13.12	9.91	2.31	5	20.33	18.06
		Aduthurai	5.36	15.21	9.81	4.12	3.20	25.00	9.67
5	FLL	New Delhi	38.72	39.14	42.23	4.05	23.12	78.20	99.67
		Karnal	31.23	30.51	32.85	2.22	21.62	52.54	92.58
		Aduthurai	37.38	34.90	37.55	2.87	20.94	57.38	84.84
6	FLW	New Delhi	2.53	1.81	2.12	0.08	1.16	3.28	31.61
		Karnal	2.4	1.75	2.07	0.37	1.26	3.12	31.61
		Aduthurai	1.74	1.39	1.59	0.16	0.88	2.58	52.9
7	SPP	New Delhi	396	218	285	37	130	509	20.00
		Karnal	410	186	273	40	117	504	9.35
		Aduthurai	284	156	224	36	82	471	41.61
8	FGP	New Delhi	247	170	191	24	13	332	48.71
		Karnal	260	157	202	27	83	377	29.03
		Aduthurai	171	139	167	26	49	369	75.48
9	PSTE	New Delhi	37.03	21.60	31.96	5.94	5.00	95.00	68.38
		Karnal	36.62	15.05	25.05	5.02	5.47	73.13	46.45
		Aduthurai	37.97	11.47	23.69	7.92	6.58	70.99	32.25
10	TGW	New Delhi	17.42	25.75	21.76	0.60	13.53	30.02	21.29
		Karnal	18.15	26.81	22.32	0.51	15.83	32.45	7.74
		Aduthurai	18.10	26.95	22.87	0.94	16.83	33.46	11.61
11	YLD	New Delhi	30.38	45.29	34.19	7.01	4.59	59.78	55.16
		Karnal	26.31	42.12	33.91	6.68	9.22	60.73	30.32
		Aduthurai	9.50	31.35	19.45	6.51	3.10	44.97	14.52
12	SSD	New Delhi	15.79	7.66	10.98	1.33	4.75	20.23	8.06
		Karnal	18.45	7.03	11.17	1.68	5.13	23.49	3.87
		Aduthurai	12.62	6.12	9.40	1.58	3.68	18.97	23.87

**Table 2:** Mean sum of squares of yield related traits obtained by combined analysis over three locations

**Source**	**Degrees of freedom**	**DFF**	**PHT**	**PL**	**PPP**	**FLL**	**FLW**	**SPP**	**FGP**	**PSTE**	**TGW**	**YLD**	**SSD**
location	2	3087**	26714**	159044**	3236**	7036**	89311**	325297**	94302**	6017**	97**	22774**	294**
Blocks (loc)	12	2.94*	24.73**	1.70^NS^	3.43**	12.63**	4.57**	651.43^NS^	686.52**	120.68**	0.46**	170.90**	0.83^NS^
RILs	313	126.92**	113.76**	43.64**	7.17**	82.22**	83.66**	11159.91**	5312.14**	406.73**	20.75**	114.04**	19.33**
loc*RILs	626	17.16**	32.55**	9.46**	1.67*	21.32**	12.23**	1827.20**	1758.94**	101.29**	1.50**	57.90**	2.57**

*Significance at 5%; **Significance at 1%.

In Additive Main effects and Multiplicative Interaction effects (AMMI) analyses among twelve traits, the first two components explained most of the variation (~100%) of G × E interaction effect, of which component1 explained more than 75% of G × E variation for days to 50% flowering, plant height and flag leaf length and for remaining traits it explained G × E in the range of 61.9-69.8%. Component 2 explained the G × E interaction variation in the range of 22.4-38.0% for the traits under study (Table 3).

**Table 3:** Percentage of variation explained by first two principal components of AMMI model

**Principal components**	**DFF**	**PHT**	**PL**	**PPP**	**FLL**	**FLW**	**SPP**	**FGP**	**PSTE**	**TGW**	**YLD**	**SSD**
PC1	77.6	75.2	63.2	63.8	75.6	69.8	61.9	67.3	67.1	68.7	67.3	64.5
PC2	22.4	24.8	36.8	36.2	24.4	30.2	38.1	32.7	32.9	31.3	32.7	35.5

### Correlation coefficients between yield and yield related traits

A total of 66 pair wise combinations were formed among twelve traits of which 56 coefficients of correlation estimates were found to be significant at 1% level of significance and three combinations were significant at 5% level of significance (Table 4). Over all the significant correlation coefficients among yield components and between yield and yield components for majority of the character pairs indicated sufficient variation in the RILs population under study indicating that the RIL population was appropriate for QTL mapping studies.

**Table 4:** **Correlations of the yield and yield related traits in Pusa1266 X Jaya RIL population in *Kharif,* 2006**

**Trait**	**DFF**	**PHT**	**PL**	**PPP**	**FLL**	**FLW**	**SPP**	**FGP**	**PSTE**	**TGW**	**YLD**	**SSD**
DFF	1.00											
PHT	−0.07*	1.00										
PL	0.01^NS^	0.62**	1.00									
PPP	−0.10**	−0.01^NS^	0.09**	1.00								
FLL	0.10**	0.29**	0.43**	0.07*	1.00							
FLW	0.22**	0.53**	0.23**	−0.29**	0.1**	1.00						
SPP	0.14**	0.41**	0.29**	−0.28**	0.3**	0.56**	1.00					
FGP	−0.09**	0.36**	0.19**	−0.26**	0.0^NS^	0.41**	0.70**	1.00				
PSTE	0.29**	0.06*	0.12**	−0.04 ^NS^	0.4**	0.20**	0.35**	−0.39**	1.00			
TGW	−0.16**	0.05 ^NS^	0.13**	0.16**	−0.1**	−0.17**	−0.24**	−0.13**	−0.15**	1.00		
YLD	−0.25**	0.51**	0.35**	0.36**	0.0 ^NS^	0.29**	0.34**	0.53**	−0.25**	0.16**	1.00	
SSD	0.14**	0.24**	−0.02 ^NS^	−0.31**	0.2**	0.51**	0.95**	0.68**	0.33**	−0.30**	0.26**	1.00

### QTL mapping by composite interval mapping

**Figure 2:**
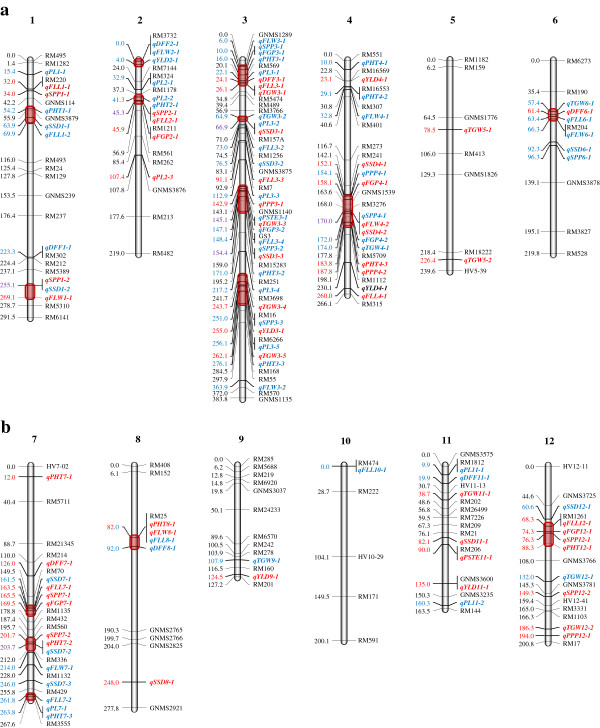
**Genetic linkage map of rice chromosomes 1-12 on the Pusa1266XJaya recombinant inbred line population along with peak positions of quantitative trait loci (QTLs) for twelve traits of rice.** QTL hotspots are denoted by brown color boxes on linkage groups. QTLs contributed by Pusa1266 and Jaya are denoted by red and blue colors respectively.

QTL mapping in the present experiment was carried out by calculating threshold logarithm of odds (LOD) for each trait by performing test with 1000 permutations. The experimental threshold LOD mean were 2.96 and 3.69 at 5% and 1% level of significance respectively. The threshold levels varied for different traits in three locations. A total of 112 QTLs affecting 12 traits spread over 12 linkage groups were identified, of these 11 QTLs were detected across three locations and 23 QTLs across two locations (Table 5, Figure 2). The number of QTLs per trait ranged from a minimum of two QTLs for number of per cent sterility to 14 for flag leaf length and spikelet setting density. QTLs affecting flag leaf length were distributed on nine chromosomes followed by spikelet setting density on eight chromosomes, whereas, per cent sterility had QTLs on two chromosomes. Five traits, namely days to 50% flowering, plant height, flag leaf width, spikelets per panicle and 1000-grain weight had QTLs on seven chromosomes. Results with respect to trait wise QTLs identified are presented below.

**Table 5:** Yield and yield related QTLs detected in Pusa1266 × Jaya RIL population across three locations in India

**Sl.No**	**QTL**	**Chr**	**Flanking Marker interval**	**Peak point**	**Increasing effect**	**New Delhi**		**Karnal**		**Aduthurai**	
						**LOD**	**R**^**2**^	**LOD**	**R**^**2**^	**LOD**	**R**^**2**^
Days to 50% flowering											
1	*qDFF1-1*	1	RM302-RM212	223.3	Jaya	-	-	-	-	3.58**	0.04
2	*qDFF2-1*	2	RM3732-RM7144	0.0	Jaya	-	-	-	-	3.44**	0.04
3	*qDFF3-1*	3	GNMS1289-RM5474	24.1	Jaya	16.2**	0.18	20.3**	0.25	-	-
4	*qDFF6-1*	6	RM190-GNMS3878	61.4	Pusa1266	14.3**	0.25	7.59**	0.1	5.96**	0.11
5	*qDFF7-1*	7	RM214-RM70	126	Jaya	-	-	5.95**	0.15	-	-
6	*qDFF8-1*	8	RM25-GNMS2765	92	Pusa1266	22.9**	0.42	16**	0.41	5.89**	0.07
7	*qDFF11-1*	11	GNMS3575-HV11-13	19.9	Pusa1266	-	-	-	-	4.14**	0.07
Plant height											
8	*qPHT1-1*	1	GNMS114-GNMS3879	54.2	Pusa1266	3.72**	0.05	4.23**	0.06	-	-
9	*qPHT2-1*	2	RM1178-RM1211	45.3	Jaya	-	-	-	-	3.96**	0.05
10	*qPHT3-1*	3	GNMS1289-RM569	16.01	Jaya	-	-	-	-	7.62**	0.11
11	*qPHT3-2*	3	RM15283-RM251	171.01	Jaya	3.59**	0.1	-	-	-	-
12	*qPHT3-3*	3	RM6266-RM168	276.1	Jaya	-	-	3.04*	0.07	-	-
13	*qPHT4-1*	4	RM551-RM16569	10.01	Jaya	3.44*	0.07	-	-	-	-
14	*qPHT4-2*	4	RM16553-RM307	29.1	Jaya	4.89**	0.06	4.05**	0.05	-	-
15	*qPHT4-3*	4	RM5709-RM1112	183.8	Pusa1266	4.35**	0.09	4.81**	0.09	-	-
16	*qPHT7-1*	7	HV7-02-RM5711	12.01	Pusa1266	-	-	3.83**	0.11	-	-
17	*qPHT7-2*	7	RM560-RM336	203.7	Jaya	-	-	-	-	3.65*	0.06
18	*qPHT7-3*	7	RM429-RM3555	263.8	Pusa1266	-	-	3.43*	0.05	-	-
19	*qPHT8-1*	8	RM25-GNMS2765	82.01	Pusa1266	3.09*	0.04	-	-	-	-
20	*qPHT12-1*	12	RM1261-GNMS3766	88.31	Jaya	-	-	-	-	4.56**	0.14
Panicle length											
21	*qPL1-1*	1	RM1282-RM220	15.41	Pusa1266	3*	0.07	-	-	-	-
22	*qPL2-1*	2	RM3732-RM324	32.91	Jaya	-	-	6.7**	0.08	-	-
23	*qPL2-2*	2	RM1178-RM1211	41.3	Jaya	-	-	6.17**	0.08	4.87**	0.07
24	*qPL2-3*	2	RM262-GNMS3876	107.4	Jaya	7.74**	0.1	-	-	-	-
25	*qPL3-1*	3	RM569-RM5474	22.11	Jaya	3.22*	0.05	-	-	9.06**	0.12
26	*qPL3-2*	3	RM3766-RM157A	66.9	Jaya	3.57*	0.05	3.96**	0.06	-	-
27	*qPL3-3*	3	RM7-GNMS1140	112.9	Jaya	5.95**	0.22	-	-	-	-
28	*qPL3-4*	3	RM15283-RM3698	217.2	Jaya	5.18**	0.22	-	-	-	-
29	*qPL3-5*	3	RM16-RM168	256.1	Jaya	-	-	3.37*	0.04	-	-
30	*qPL7-1*	7	RM429-RM3555	263.8	Pusa1266	-	-	3.87**	0.05	-	-
31	*qPL11-1*	11	RM1812-HV11-13	9.9	Pusa1266	-	-	-	-	3.52*	0.04
32	*qPL11-2*	11	GNMS3235-RM144	160.3	Jaya	-	-	3.7**	0.05	-	-
Panicles per plant											
33	*qPPP3-1*	3	RM7-GNMS1140	142.9	Pusa1266	3.02**	0.04	-	-	-	-
34	*qPPP4-1*	4	RM273-GNMS1539	154.11	Jaya	5.73**	0.13	4.81**	0.09	-	-
35	*qPPP4-2*	4	RM3276-RM1112	187.8	Jaya	7.11**	0.10	5.18**	0.08	9.74**	0.19
36	*qPPP12-1*	12	RM1103-RM17	194	Jaya	-	-	3.16*	0.06	-	-
Flag leaf length											
37	*qFLL1-1*	1	RM220-GNMS114	32.01	Jaya	-	-	3.75**	0.04	-	-
38	*qFLL1-2*	1	GNMS3879-RM493	69.91	Jaya	3.3*	0.10	-	-	-	-
39	*qFLL2-1*	2	RM324-RM1211	45.31	Jaya	3.03*	0.04	3.73**	0.05	-	-
40	*qFLL3-1*	3	GNMS1289-RM5474	26.11	Jaya	-	-	-	-	5.93**	0.1
41	*qFLL3-2*	3	RM157A-RM1256	73.01	Jaya	-	-	3.02*	0.04	-	-
42	*qFLL3-3*	3	GNMS3875-RM7	91.11	Jaya	-	-	3.07*	0.04	-	-
43	*qFLL3-4*	3	GS3-RM15283	148.4	Pusa1266	-	-	-	-	2.99*	0.04
44	*qFLL4-1*	4	RM1112-RM315	260	Jaya	-	-	3.52**	0.07	3.05*	0.22
45	*qFLL6-1*	6	RM190-RM204	63.4	Pusa1266	7.92**	0.12	2.92*	0.07	-	-
46	*qFLL7-1*	7	RM70-RM1135	163.5	Pusa1266	-	-	4.73**	0.13	-	-
47	*qFLL7-2*	7	RM429-RM3555	261.8	Pusa1266	3.3*	0.05	-	-	-	-
48	*qFLL8-1*	8	RM25-GNMS2765	82.01	Pusa1266	6.79**	0.08	-	-	-	-
49	*qFLL10-1*	10	RM474-RM222	0.0	Pusa1266	-	-	-	-	3.11*	0.04
50	*qFLL12-1*	12	RM1261-GNMS3766	68.31	Jaya	2.9*	0.03	-	-	-	-
Flag leaf width											
51	*qFLW1-1*	1	RM5389-RM6141	269.1	Pusa1266	4.22**	0.07	-	-	-	-
52	*qFLW2-1*	2	RM3732-RM7144	0.0	Jaya	-	-	-	-	4.26**	0.04
53	*qFLW3-1*	3	GNMS1289-RM569	6.01	Jaya	-	-	-	-	4.02**	0.05
54	*qFLW3-2*	3	RM55-RM570	363.9	Pusa1266	-	-	3.1*	0.04	3.44**	0.18
55	*qFLW4-1*	4	RM307-RM401	32.81	Jaya	3.82**	0.04	-	-	-	-
56	*qFLW4-2*	4	RM3276-RM5709	170.01	Pusa1266	40**	0.44	46.5**	0.53	25.9**	0.33
57	*qFLW6-1*	6	RM204-GNMS3878	66.31	Pusa1266	2.87*	0.02	-	-	-	-
58	*qFLW7-1*	7	RM336-RM1132	214.01	Jaya	2.89*	0.03	-	-	-	-
59	*qFLW8-1*	8	RM25-GNMS2765	82.01	Pusa1266	2.92*	0.02	-	-	-	-
Spikelets per panicle											
60	*qSPP1-1*	1	RM220-GNMS114	34.01	Jaya	-	-	3.15*	0.04	-	-
61	*qSPP1-2*	1	RM5389-RM5310	255.1	Pusa1266	3.31*	0.11	-	-	-	-
62	*qSPP2-1*	2	RM324-RM1211	45.31	Jaya	-	-	-	-	4.56**	0.05
63	*qSPP3-1*	3	GNMS1289-RM5474	10.01	Jaya	-	-	-	-	4.18**	0.07
64	*qSPP3-2*	3	GNMS1140-RM15283	154.4	Pusa1266	-	-	-	-	11.4**	0.15
65	*qSPP3-3*	3	RM3698-RM168	251.01	Pusa1266	3.39*	0.04	-	-	-	-
66	*qSPP4-1*	4	RM3276-RM5709	170.01	Pusa1266	6.58**	0.09	13.8**	0.16	9.42**	0.13
67	*qSPP6-1*	6	RM204-GNMS3878	96.31	Pusa1266	3.42*	0.3	-	-	-	-
68	*qSPP7-1*	7	RM70-RM432	165.5	Pusa1266	3.53*	0.09	11.4**	0.21	-	-
69	*qSPP7-2*	7	RM560-RM336	201.71	Pusa1266	3.93**	0.07	6.25**	0.09	-	-
70	*qSPP12-1*	12	RM1261-GNMS3766	76.31	Jaya	3.02*	0.07	-	-	4.35**	0.08
71	*qSPP12-2*	12	GNMS3781-HV12-41	149.31	Jaya	-	-	4.03**	0.05	-	-
Filled grains per panicle											
72	*qFGP2-1*	2	RM1211-RM561	45.91	Jaya	-	-	-	-	4.04**	0.05
73	*qFGP3-1*	3	GNMS1289-RM569	10.01	Jaya	-	-	-	-	3.53**	0.06
74	*qFGP3-2*	3	GNMS1140-GS3	147.11	Jaya	3.43*	0.05	2.89*	0.03	-	-
75	*qFGP4-1*	4	RM241-GNMS1539	158.11	Pusa1266	7.5**	0.15	12.8**	0.21	-	-
76	*qFGP4-2*	4	RM3276-RM5709	172.0	Pusa1266	9.38**	0.15	15.3**	0.22	8.17**	0.12
77		7	RM70-RM1135	169.51	Pusa1266	-	-	6.32**	0.13	-	-
78	*qFGP12-1*	12	RM1261-GNMS3766	74.31	Jaya	-	-	-	-	4.11**	0.07
Per cent sterility											
79	*qPSTE3-1*	3	RM7-GS3	145.11	Pusa1266	14.24**	0.18	10.00**	0.18	7.85**	0.18
80	*qPSTE11-1*	11	RM206-GNMS3600	90.0	Pusa1266	-	-	2.8*	0.03	-	-
1000 grain weight											
81	*qTGW3-1*	3	GNMS1289-RM5474	26.11	Jaya	-	-	-	-	8.08**	0.13
82	*qTGW3-2*	3	RM3766-RM157A	64.91	Jaya	-	-	3.33*	0.05	-	-
83	*qTGW3-3*	3	GNMS1140-RM15283	145.11	Pusa1266	-	-	5.23**	0.06	-	-
84	*qTGW3-4*	3	RM3698-RM16	243.71	Pusa1266	-	-	-	-	5.42**	0.07
85	*qTGW3-5*	3	RM6266-RM168	262.11	Pusa1266	-	-	-	-	6.08**	0.1
86	*qTGW4-1*	4	RM3276-RM1112	174.01	Jaya	-	-	4.76**	0.07	3.81**	0.05
87	*qTGW5-1*	5	GNMS1776-RM413	78.51	Jaya	-	-	4.93**	0.18	5.11**	0.2
88	*qTGW5-2*	5	GNMS1826-HV5-39	226.41	Jaya	4.78**	0.09	3.22*	0.26	-	-
89	*qTGW6-1*	6	RM6273-RM204	57.41	Jaya	12.9**	0.25	6.39**	0.14	6.42**	0.16
90	*qTGW9-1*	9	RM278-RM160	107.91	Pusa1266	-	-	2.92*	0.04	-	-
91	*qTGW11-1*	11	HV11-13-RM202	38.71	Jaya	-	-	2.83*	0.05	-	-
92	*qTGW12-1*	12	RM1261--GNMS3781	132.01	Pusa1266	-	-	2.77*	0.07	-	-
93	*qTGW12-2*	12	RM1103-RM17	186.31	Pusa1266	4.23**	0.1	7.22**	0.18	3.03*	0.07
Yield per plant											
94	*qYLD2-1*	2	RM3732-RM7144	4.01	Jaya	-	-	-	-	3.24*	0.05
95	*qYLD3-1*	3	RM16-RM168	255.01	Pusa1266	-	-	-	-	3.6*	0.04
96	*qYLD4-1*	4	RM1112-RM315	23.11	Jaya	-	-	-	-	3.28*	0.16
97	*qYLD9-1*	9	RM160-RM201	124.51	Jaya	6.88**	0.11	-	-	-	-
98	*qYLD11-1*	11	GNMS3600-GNMS3235	135.01	Jaya	-	-	-	-	4.51**	0.05
Spikelet setting density											
99	*qSSD1-1*	1	GNMS3879-RM493	63.91	Jaya	3.37**	0.07	-	-	-	-
100	*qSSD1-2*	1	RM5389-RM5310	255.11	Pusa1266	2.82*	0.09	-	-	-	-
101	*qSSD3-1*	3	RM3766-RM157A	66.91	Pusa1266	2.95*	0.04	-	-	-	-
102	*qSSD3-2*	3	RM1256-GNMS3875	76.51	Pusa1266	3.32*	0.04	-	-	-	-
103	*qSSD3-3*	3	GS3-RM15283	154.41	Pusa1266	-	-	-	-	11.7**	0.16
104	*qSSD4-1*	4	RM273-GNMS1539	152.11	Pusa1266	10.2**	0.2	15**	0.23	10.2**	0.17
105	*qSSD4-2*	4	RM3276-RM5709	170.01	Pusa1266	11.4**	0.14	16.3**	0.19	12.4**	0.17
106	*qSSD6-1*	6	RM204-GNMS3878	92.31	Pusa1266	3.71**	0.25	3.44**	0.1	-	-
107	*qSSD7-1*	7	RM70-RM1135	161.51	Pusa1266	-	-	10.2**	0.19	3.95*	0.09
108	*qSSD7-2*	7	RM560-RM336	203.71	Pusa1266	4.96**	0.08	7.23**	0.10	-	-
109	*qSSD7-3*	7	RM1132-RM429	246.01	Pusa1266	-	-	3.48**	0.05	-	-
110	*qSSD8-1*	8	GNMS2825-GNMS2921	248.01	Jaya	-	-	-	-	2.99*	0.22
111	*qSSD11-1*	11	RM21-206	82.11	Pusa1266	-	-	2.87*	0.03	-	-
112	*qSSD12-1*	12	GNMS3725-RM1261	60.61	Jaya	-	-	-	-	3.75*	0.06

*Significance at 5%; **Significance at 1%.

#### *Days to 50*% *flowering*

Seven QTLs affecting days to 50% flowering (DFF) were identified on seven different chromosomes. Of these, two QTLs were identified across three locations, one QTL across two locations and four QTLs at specific locations. Out of seven QTLs identified, three loci were contributed by the parent Pusa1266 whereas, Jaya contributed four QTLs. The QTL *qDFF8-1* on chromosome 8 at marker interval RM25-GNMS2765, was identified at 1% level of significance in three locations with a LOD value range of 5.89-22.9 explaining 7-42% of phenotypic variation. Another QTL *qDFF6-1* was identified across three locations with LOD value of 5.96-14.3 explaining phenotypic variation up to 25%. At both QTL positions, Pusa1266 allele contributed increasing effect.

#### *Plant height*

Plant height (PHT) in Pusa1266 X Jaya RIL population was influenced by 13 genomic regions on seven chromosomes of which three QTLs were identified over two locations and ten were location specific. Out of thirteen QTLs identified, five were contributed by Pusa1266 and eight by parent Jaya. A QTL *qPHT1-1* was identified at New Delhi and Karnal at the marker interval of GNMS114-GNMS3879 with LOD value range of 3.72-4.23 explaining 5-7% of phenotypic variation. Among location specific QTLs, *qPHT3-3, qPHT7-1, qPHT12-1*, were identified at New Delhi, Karnal and Aduthurai Explaining 10%, 11% and 14% of phenotypic variation respectively.

#### *Panicle length*

Panicle length (PL) was governed by 12 QTLs dispersed on 5 chromosomes. Among them, three QTLs expressed over two locations and nine QTLs were location specific. The parent Jaya contributed enhanced effect at 9 QTLs whereas Pusa1266 made positive contribution at three QTL positions. A QTL *qPL3-1* was identified across two locations i.e., New Delhi and Aduthurai at marker interval RM569-RM5474 with LOD value range of 3.22-9.06 explaining 5-12% of phenotypic variation with Jaya allele contributing increased the phenotypic effect at this locus. At New Delhi two major QTLs i.e., *qPL3-3*, *qPL3-4* were identified at LOD value >5.0 explaining 22% of phenotypic variation each.

#### *Number of panicles per plant*

Four QTLs were identified on three chromosomes that influenced number of panicles per plant (PPP). Among them, one QTL on chromosome 4 expressed across three environments, one QTL expressed across two environments and two QTLs were specific to one location. The Jaya allele contributed to trait increase at three loci whereas Pusa1266 contributed at one locus. A QTL *qPPP4-2* at marker interval RM3276-RM1112 was identified across three locations with a LOD value range of 5.18-9.74 explaining 8-19% of phenotypic variation.

#### *Flag leaf length*

Flag leaf length (FLL) was influenced by 14 QTLs spread over 9 chromosomes of which, three QTLs were detected across two environments and eleven QTLs were location specific. The parent Jaya contributed positive allele at 8 QTL positions while Pusa1266 contributed 6 positive alleles. A QTL *qFLL4-1* expressed across two environments was identified with LOD value range of 3.05-3.52 explaining 7-22% of phenotypic variation. A novel QTL *(qFLL7-1*) was identified at Karnal with a LOD 4.73 explaining 13% of phenotypic variation.

#### *Flag leaf width*

Out of nine QTLs controlling flag leaf width (FLW), *qFLW4-2* was identified across three environments, another QTL *qFLW3-2* was identified at Karnal and Aduthurai and remaining seven QTLs were location specific. Pusa1266 contributed positive effect at five loci and Jaya allele at four loci. A major robust QTL *qFLW4-2* was identified across three locations at marker interval RM3276-RM5709 with LOD value range of 21.69-46.49, explaining 33-53% of phenotypic variation.

#### *Spikelets per panicle*

A total of twelve QTLs on seven chromosomes affecting spikelets per panicle (SPP) were identified. Among them, two QTLs expressed across three environments, two across two environments and eight were location specific. The Pusa1266 allele contributed to trait increase at seven loci and the Jaya allele at five loci. A major QTL *qSPP4-1* in the marker interval of RM3276-RM5709 was identified across three locations with LOD 6.58-13.84 explaining 9-16% phenotypic variation. The Pusa1266 allele increased 20.14-28.61 spikelets at this locus. A major novel QTL, *qSPP6-1*, was identified at New Delhi with LOD 3.42 explaining 30% of phenotypic variation.

#### *Filled grains per panicle*

Filled grains per panicle (FGP) were influenced by seven QTLs on five chromosomes. Out of 7 QTLs influencing the trait, one expressed across three locations, two across two locations and four were specific to one location. In contrast to other traits, higher phenotypic value parent Pusa1266 contributed lesser number of QTLs (3) than lower phenotypic value parent Jaya (4). A major QTL, *qFGP4-2* was identified at marker interval RM3276-RM5709 expressing across three locations with LOD values 8.17-15.27, explaining 12-22% of phenotypic variation. A QTL specific to Karnal *qFGP7-1* was identified with LOD 6.32 explaining 13% of phenotypic variation.

#### *Per cent sterility*

Per cent sterility (PSTE) was influenced by two QTLs on two chromosomes. Of which, one QTL expressed across three locations and other QTL was location specific and the Pusa1266 allele contributed positive effect at all loci. The QTL *qPSTE3-1* in the marker interval RM7-RM15283 was identified at LOD value range of 7.85-14.24, explaining 18% of phenotypic variation.

#### *1000 grain weight*

Thirteen QTLs influencing 1000 grain weight (TGW) were identified in the present study. Out of which, two QTLs expressed across three locations, three across two locations and eight were location specific. The Jaya allele contributed increased effect at seven QTLs and the Pusa1266 allele at six QTL positions. Among two QTLs expressing across three locations, a novel QTL *qTGW6-1* was identified with LOD value ranging from 6.42-12.9, which explained maximum phenotypic variation (5-31%). Among QTLs expressing across two locations, QTL *qTGW5-1* was identified at Karnal and Aduthurai with LOD value ranging from 4.93-6.12 explaining ~20% of phenotypic variation. A novel QTL specific to Aduthurai, *qTGW3-1,* was identified at LOD 8.08 explaining 13% of phenotypic variation.

#### *Grain yield per plant*

A total of 5 QTLs were identified for grain yield per plant (YLD). Among these four QTLs were identified at Aduthurai and one QTL at New Delhi. Out of five QTLs, the Jaya allele caused increased effect at four loci and the Pusa1266 allele contributed increased effect at one QTL position. A major QTL, *qYLD4-1*, was identified at Aduthurai with LOD 3.28 explaining 15% of phenotypic variation. At New Delhi, a location specific QTL *qYLD9-1* at marker interval RM160-RM201 with LOD 6.88 and explaining 11% of phenotypic variation was identified.

#### *Spikelet setting density*

Fourteen QTLs on eight chromosomes influenced spikelet setting density (SSD). Of these QTLs, two expressed across three locations, three across two locations and nine at one location. The parent Pusa1266 with higher phenotypic value contributed increased effect at 11 QTL positions whereas the Jaya allele contributed increased effect at three QTLs. Two novel QTLs, *qSSD4-1* and *qSSD4-2*, expressing across three locations were identified at LOD 10.2-15 and 11.4-16.3 explaining 17-23% and 17-19% of phenotypic variation respectively. A location specific QTL, qSSD3-3, was identified at Aduthurai with LOD 11.70 explaining 16% of phenotypic variation.

### Novel QTLs

Of the 112 QTLs identified in the study, 44 QTLs were found to be novel. Eleven out of twelve traits had at least one novel QTL (Table 6). Pusa1266, a new plant type *indica*/*japonica* derivative contributed nineteen novel QTLs whereas Jaya contributed twenty five novel QTLs. Maximum number of novel QTLs were found for flag leaf length (10) followed by 1000-grain weight, spikelet setting density (6 each), plant height (5), panicle length (4), flag leaf width, spikelets per panicle, grain yield per plant (3 each), filled grains per panicle (2) and panicles per plant, per cent sterility (1 each). The novel QTLs were present on all chromosomes except chromosomes 5, 9 and 10. Maximum number of novel QTLs (10) were identified on chromosome 3 followed by chromosome 2 (7). Out of 44 novel QTLs, four QTLs expressing across three locations, nine QTLs expressing across two locations and thirty one location specific QTLs were identified. Two novel QTLs i.e., *qTGW6-1*, *qTGW12-2* expressing across three environments were identified for 1000-grain weight with LOD value of 12.9 explaining 25% of phenotypic variation. For spikelet setting density, two novel QTLs *qSSD4-1*, *qSSD4-2* expressing across three locations were identified with a LOD value of 15 explaining up to 23% of phenotypic variation.

**Table 6:** Novel QTLs identified in Pusa1266 X Jaya RIL Population

**Sl. No**	**Trait Name**	**No of QTLs**	**QTL Name**	**Three Locations**	**Two locations**	**One location**
1	PHT	5	*qPHT2-1, qPHT3-3, qPHT4-1, qPHT4-2, qPHT12-1*	-	1	4
2	PL	4	*qPL2-2, qPL2-3, qPL3-1, qPL3-5*	-	2	2
3	PPP	1	*qPPP3-1*	-	-	1
4	FLL	10	*qFLL2-1, qFLL3-1, qFLL3-2, qFLL3-3, qFLL3-4, qFLL6-1, qFLL7-1, qFLL7-2, qFLL8-1, qFLL12-1*	-	2	8
5	FLW	3	*qFLW1-1, qFLW4-1, qFLW8-1*	-	-	3
6	SPP	3	*qSPP2-1, qSPP6-1, qSPP12-2*	-	-	3
7	FGP	2	*qFGP2-1, qFGP12-1*	-	-	2
8	PSTE	1	*qPSTE11-1*	-	-	1
9	TGW	6	*qTGW3-1, qTGW4-1, qTGW6-1, qTGW11-1, qTGW12-1, qTGW12-2*	2	1	3
10	YLD	3	*qYLD2-1, qYLD3-1, qYLD11-1*	-	-	3
11	SSD	6	*qSSD1-2, qSSD4-1, qSSD4-2, qSSD6-1, qSSD7-1, qSSD7-2*	2	3	1
	Total	44		4	9	31

Nine novel QTLs expressing across two environments were identified for five traits namely plant height (1), panicle length (2), flag leaf length (2), 1000-grain weight (1) and spikelet setting density (3). A novel QTL *qPL3-1* was identified at New Delhi and Aduthurai with a LOD value ranging from 3.22-9.06 explaining up to 12% of phenotypic variation. For flag leaf length, a novel QTL *qFLL6-1* was identified at New Delhi and Karnal with LOD value of 7.92 explaining up to 12% of phenotypic variation. Three novel QTLs expressing across two environments were identified for spikelet setting density, of which a major QTL *qSSD6-1* expressing at New Delhi and Karnal was identified with a LOD value of 3.71 explaining up to 25% of phenotypic variation. A major novel QTL *qSPP6-1* expressing at New Delhi was identified with a LOD value of 3.42 explaining 30% of phenotypic variation. A location specific QTL for plant height *qPHT12-1* expressing at Aduthurai was identified with a LOD value of 4.56 explaining 14% of phenotypic variation. For 1000 grain weight, a novel QTL *qTGW3-1* was identified at Aduthurai with a LOD value of 8.08 explaining 13% of phenotypic variation.

### QTL Hotspots

Fifteen QTL hotspots (clusters) were identified on eight chromosomes often for correlated traits such as plant height, panicle length, flag leaf, spikelets per panicle, filled grains per panicle and spikelet setting density (Table 7). Maximum QTL hotspots (4) were identified on chromosome 3 and a minimum of one QTL hotspot on chromosomes 4, 6, 8 and 12. Four QTL hot spots were observed on chromosomes 1 (I, II) and 2 (III and IV) harboring three to four QTLs each influencing plant height, panicle length, flag leaf traits, spikelets per panicle and spikelet setting density in a window size around ~15 cM. On chromosome 3, four QTL hotspots (V-VIII) were observed of which, two clusters (V and VII) are particularly interesting. QTL cluster V and VII are harboring seven to eight QTLs affecting either closely or allometrically related traits. Two robust QTL hotspots expressing across locations were identified on chromosome 4 (IX) and 6 (X) influencing important yield component traits i.e., flag leaf length and width, panicles per plant, spikelets and grains per panicle, thousand grain weight and spikelet setting density. Eleven QTLs affecting plant height, flag leaf length and width, spikelet and filled grains per panicle were found in three clusters (XI, XII and XIII) on chromosome seven in a window size ranging from 2–12 cM. One QTL hotpot each on chromosome 8 (XIV) and 12 (XV) harboring four QTLs in each cluster affecting traits like plant height, flag leaf length and width, spikelets per panicle and filled grains per panicle.

**Table 7:** QTL hotspots identified in Pusa1266 X Jaya RIL Population

**Sl. No**	**QTL Cluster No.**	**Chromosome**	**Peak interval (cM)**	**Window Size (cM)**	**No of QTL**	**Name of QTL**
1	I	1	54.2-69.9	15.7	3	*qPHT1-1, qSSD1-1, qFLL1-2*
2	II	1	255.1-269.1	14.0	3	*qSPP1-2, qSSD1-2, qFLW1-1*
3	III	2	0.0-4.0	4.0	3	*qDFF2-1, qFLW2-1, qYLD2-1*
4	IV	2	41.3-45.3	4.0	4	*qPL2-2, qPHT2-1, qSPP2-1, qFLL2-1*
5	V	3	6.0-26.1	20.1	8	*qFLW3-1, qSPP3-1, qFGP3-1, qPHT3-1, qPL3-1, qDFF3-1, qFLL3-1, qTGW3-1*
6	VI	3	64.9-66.9	2.0	3	*qTGW3-2, qPL3-2, qSSD3-1*
7	VII	3	142.9-154.4	11.5	7	*qPPP3-1, qSTE3-1, qTGW3-3, qFGP3-2, qFLL3-4, qSPP3-2, qSSD3-3*
8	VIII	3	243.7-262.1	18.4	5	*qTGW3-4, qSPP3-3, qYLD3-1, qPL3-5, qTGW3-5*
9	IX	4	154.1-174.0	20	7	*qPPP4-1, qFGP4-1, qSPP4-1, qFLW4-2, qSSD4-2, qFGP4-2, qTGW4-1*
10	X	6	57.4-66.3	8.9	4	*qTGW6-1, qDFF6-1, qFLL6-1, qFLW6-1*
11	XI	7	161.5-169.5	8.0	4	*qSSD7-1, qFLL7-1, qSPP7-1, qFGP7-1*
12	XII	7	201.7-214.0	12.3	4	*qSPP7-2, qPHT7-2, qSSD7-2, qFLW7-1*
13	XIII	7	261.8-263.8	2.0	3	*qFLL7-2, qPL7-1, qPHT7-3*
14	XIV	8	82.0-92.0	10	4	*qPHT8-1, qFLW8-1, qFLL8-1, qDFF8-1*
15	XV	12	68.3-88.3	20	4	*qFLL12-1, qFGP12-1, qSPP12-1, qPHT12-1*
Total					66	

### Epistatic QTLs

A total of 6 epistatic QTLs affecting four traits were identified. Of these, five QTLs affecting three traits were identified at New Delhi and one at Aduthurai (Table 8). For panicle length, a QTL at marker interval RM262-GNMS3876 on chromosome 2 showed significant interaction with two QTLs on chromosome 3 i.e. RM7-GNMS1140 (LOD = 3.1 R^2^ = 6.1%) and RM251-RM3698 (LOD = 1.8 R^2^ = 6.9%). Two major epistatic QTLs were identified for spikelet setting density, showing interaction between genomic locations on chromosome 1 at marker interval RM5389-RM5310 and chromosome 6 at RM190-GNMS3878 explaining >20% phenotypic variation. Whereas at Aduthurai, interaction between two marker intervals on different chromosomes i.e. RM7-GNMS1140 on chromosome 3 and RM70-RM1135 on chromosome 7 was identified with LOD 4.83 explaining 7.5% phenotypic variation.

**Table 8:** Epistatic QTLs identified in Pusa1266 X Jaya RIL Population

**New Delhi**								
**Sl. No**	**Trait**	**Chro**	**Marker Interval**	**Chro**	**Marker Interval**	**LOD**	**Additive**	**R**^**2**^
1	PL	2	RM262-GNMS3876	3	RM7-GNMS1140	3.1	0.67	6.1
2		2	RM262-GNMS3876	3	RM251-RM3698	1.77	−0.69	6.9
3	SPP	1	RM5389-RM5310	6	RM204-GNMS3878	2.91	19.71	7.6
4	SSD	1	RM5389-RM5310	6	RM190-RM204	4.02	1.54	20.4
5		1	RM5389-RM5310	6	RM204-GNMS3878	3.16	−1.45	21.6
Aduthurai								
6	PSTE	3	RM7-GNMS1140	7	RM70-RM1135	4.83	3.8	7.5

## Discussion

The prime objective of the present study was to identify novel chromosomal regions influencing yield and yield components in a RIL population developed from cross Pusa1266, a new plant type genotype, derived from *indica* X *japonica* cross and Jaya, a popular *indica* rice variety in India. As per gramene website, a total of 2,234 QTLs were identified for yield and yield related traits through more than 50 QTL mapping studies in rice. Many researchers have used wild species like *O.nivara*, *O.rufipogon, O.glaberrima,* and *O.glumaepatula* and land races to uncover the hidden genetic variation. However, linkgae drag associated with alien gene transfer is a major bottleneck in utilizing wild species in breeding programme. In the present study, which is one of the firsts of its kind, an effort was made to identify QTLs for yield and its components from sub-species *japonica* into *indica* background using inter sub-specific variation generated by crossing a NPT line Pusa 1266 with Jaya, a widely grown *indica* rice variety. Therefore, the present investigation is a novel attempt to utilize a new plant type based recombinant inbred mapping population to identify QTLs for yield and yield related traits.

For declaring the significant association of chromosomal region with trait, thresholds levels for each trait at each location were separately computed by conducting permutation test with 1000 permutations and used for composite interval mapping. The experimental threshold LOD mean was 2.96 at 0.05 and LOD 3.69 at 0.01 level of significance. A total of 112 QTLs were detected including 11 QTLs expressing across three environments, 23 QTLs across two locations and 78 location specific QTLs. Out of 112 QTLs, 34 stable QTLs expressing across locations, were identified including at least one for each trait except yield. The phenotypic variance explained by these QTLs ranged from 5% for filled grains per panicle to 53% for flag leaf width at 0.01 level of significance showing the reliability and robustness of the QTLs identified.

The extent of complex nature of the traits was evident from the observation on number of significant QTLs per trait which ranged from two QTLs for number of per cent sterility to 14 for flag leaf length. The per cent contribution of individual QTLs to total phenotypic variation for respective traits ranged from 3 to 53% suggesting complex inheritance pattern of the traits under study. Twenty significant QTLs contributing >15% variation and expressing at two or more locations were detected. The new plant type parent Pusa1266 contributed 53 QTLs (47.3%) including most of the loci for spikelets per panicle, flag leaf width, per cent sterility and spikelet setting density whereas, Jaya contributed 59 QTLs (52.7%) for plant height, panicle length, panicles per plant, thousand grain weight and yield per plant. This establishes the importance of variation in parental allele distribution as the critical component of QTL detection and estimation of the concerned traits. The *indica* X japonica diversity would have played a significant role in accumulation of number of QTL alleles in the parent Pusa1266. Distribution of yield enhancing loci among these diverse cultivated lines would also be helpful in pyramiding of QTLs with relatively higher contribution to the value of trait related to yield.

Lack of consistency of QTLs across environments is the major hurdle for utilization of the information generated through QTL mapping experiments. In the present study twenty three QTLs expressing across two locations and eleven QTLs expressing across all three locations were identified. Such robust QTLs are important from breeding point of view.

In the present study, 68 out of 112 QTLs identified were reported earlier (9–33). To understand whether genomic regions from where genes for yield related traits were cloned, are influencing yield and its components in the present study, primers were designed for 15 genes including *sd1, Ebisud2*, *Ghd7, Hd1, Hd3, Hd6*, *PLA1, D10, TB1, HTD1*, *MOC1*, *Lax1*, *GS3, GW2* and *CKX2* and used for polymorphism survey. Of these, one marker from *GS3* region was polymorphic between Pusa1266 and Jaya. The marker from *Ghd7* region did not show amplification. Despite the presence of sufficient variation for the traits between the two parents, most of the gene based markers did not show polymorphism indicating the possibility of novel QTLs. An elaborate genotyping of RIL population with additional polymorphic markers particularly from the genomic region with poor coverage would be necessary so that other additional QTLs could be tapped. Alternatively, the failure to detect polymorphism may be due to insufficient resolution offered by the metaphor agarose. A sequence analysis of these regions may help in developing markers, which can detect QTLs in this region. The monomorphism for *sd1* based markers indicated the presence of dwarfing allele in both parents as both parents were semi dwarf in height. For days to 50% flowering, panicles per plant, spikelets per panicle and thousand grain weight one QTL each was identified from the genomic region where *Hd1, Hd3, Htd1, CKX1 and GS3* genes were cloned. At the genomic location of gene *GS3*, five QTLs i.e., *qFLL3-4*, *qSPP3-2*, *qFGP3-2*, *qPSTE3-1* and *qTGW3-3* were identified of which a one major robust QTL influencing per cent sterility explained 18% of phenotypic variation across three locations can be used to understand about sterility in *indica/ japonica* crosses.

To identify the genomic regions influencing yield component traits across different populations, the QTLs positions in the present study were compared with earlier reports and found that, 68 out of 112 QTLs identified were reported earlier [9-36]. The major stable QTLs like *qDFF8-1qPPP4-2qFLW4-2qSPP4-1qFGP4-2* and *qPSTE3-1* that mapped to the same genomic regions consistent across Oryza sp. can be directly used for marker assisted transfer of them in to widely adopted high yielding varieties.

In the present study, 44 novel QTLs were reported for the first time of which, 19 were contributed by new plant type parent Pusa 1266. All the QTLs influencing days to 50% flowering and panicles per plant shared same chromosomal regions from earlier reports indicating their possible evolutionary association in rice varieties [9-16]. Five novel QTLs were identified for plant height and all were contributed by Jaya. A novel QTL i.e., *qPHT4-2* stable at New Delhi and Karnal explaining 5-6% phenotypic variation was identified. Apparently, this locus would provide the needed diversity for the trait in rice breeding programs. Remaining eight QTLs for plant height identified in genomic regions reported earlier [17-22].

Four novel QTLs were found for panicle length in the present study and all were contributed by Jaya. Two QTLs, *qPL2-2* and *qPL3-1* expressing over two locations explaining 5-12% phenotypic variation would be much useful for improvement of panicle length of japonica lines. Eight out of twelve QTLs identified for panicle length were reported in earlier studies [5,6,10,12,23,24]. Nine novel QTLs were identified for flag leaf length of which five were contributed by Pusa1266. One novel QTL *qFLL6-1* contributed by Pusa1266 expressed at New Delhi and Karnal explaining up to 12% phenotypic variation and can be utilized for improvement of the trait at both locations. Remaining all QTLs for flag leaf length and width are in the same chromosomal regions as reported earlier [25-27]. Three novel QTLs were identified for spikelets per panicle and one QTL *qSPP6-*1 contributed by the Pusa1266 allele explaining 30% of phenotypic variation was identified at New Delhi location. Nine QTLs for spikelets per panicle and all QTLs for filled grains per panicle are reported earlier [10,18,28-33]. In the present study, QTLs for spikelet sterility were identified on chromosome 3 and 11 of which, *qPSTE3-1* is interesting as it is located near a gene influencing grain size GS3. In a recent study a QTL has been identified for percent seed set under cold water conditions at reproductive stage on chromosome 3 and used for identification of lines with high spikelet fertility under cold stress which may be different from the QTL identified in this study (34, 35). Five novel QTLs were identified for thousand grain weight of which, two QTLs *qTGW6-1* and *qTGW12-2* were stable across three locations explaining 7-25% phenotypic variation and remaining QTLs were reported in earlier studies [19,29,31-33,36]. Thousand grain weight and spikelets per panicle were two component traits of grain yield. Negative correlation between these traits has been noted by rice breeders. This could be due to increase in the one of the trait and decrease in the other trait by QTLs in the same chromosomal region.

The yield QTLs in the present study were found to be specific to either NewDelhi or Aduthurai locations, explaining up to 16% of total phenotypic variation. At Aduthurai *qYLD4-1* and at New Delhi *qYLD9-1* explained 16% and 11% of phenotypic variation, respectively may be useful in further enhancing yield of cultivars areas adapted in these regions. Of the five yield QTLs identified in the present study, three QTLs *qYLD3-1qYLD4-1* and *qYLD9-1* shared same chromosomal regions as yield QTLs reported earlier [10,12,28,31,32]. Five novel QTLs stable over two or more locations explaining 8-25% of phenotypic variation were identified for spikelet setting density. Out of 44 novel QTLs reported in the present study, twelve are stable over locations. These novel stable QTL regions are potential candidates for fine mapping and map based cloning.

We identified fifteen QTL hotspots (clusters) on eight chromosomes 1–4, 6–8 and 12 harboring QTLs traits such as plant height, panicle length, flag leaf, spikelets per panicle, filled grains per panicle and spikelet setting density. It is very interesting to examine co-locating QTLs in biological and breeding perspective while considering phenotypic and genetic correlations. One of the central concepts in genetical genomics is the existence of QTL hotspots, where a single polymorphism leads to widespread downstream changes in the expression of distant genes, which are all mapping to the same genomic locus [37]. In this study multiple QTL clusters affecting many traits were identified of which, some are either genetically correlated or allometrically related. It is very difficult to speculate the causative mechanism between all these traits in a hotspot as correlations do not suggest link between them. It is possible that these clusters represent more than one gene but the present mapping population resolution is not sufficient to differentiate whether it is due to either linkage or pleiotropy. It is observed that some hotspots contain QTLs that are not allometrically linked. It may possible that these loci represent *trans* acting QTL (most likely transcription factors) where the effect of alterations in regulation or structural characteristics would be expected to have smaller effects on many traits [38]. It can be concluded that each QTL within a QTL hotspot might only contribute a small positive effect, but co-location of multiple traits indicate that selection for beneficial allele at these loci will result in a cumulative increase in yield due to the integrative positive effect of various QTLs.

In the present study, 6 epistatic QTLs were identified for five traits with phenotypic variation ranging from 6.1% for panicle length to 21.6% for spikelet setting density. However, only limited number of epistatic interactions between QTLs were identified firstly because analysis of primary populations like RILs does not provide much information about the real nature of epistatic interactions because of the confounding effect of background loci or other QTLs with larger effect interfere with detection of interactions [39]. Secondly, in a RIL population only those QTLs showing additive x additive type of interaction could be identified, because identification of additive X dominance and dominance X dominance type epistatis is not possible owing to lack of heterozygosity.

The yield and its component traits showed a range of sensitivity response to changing environment. QTLs of traits such as plant height, flag leaf length, grain yield per plant with high environment main effect could be detected only in one or two environments. Furthermore, consistent with this observation, those genomic regions of quantitative traits with the least G X E interaction and high genotypic main effects expressed comparable phenotypes across different environments. In the present study, 78 QTLs expressing at a specific location were identified, of which 21 were major QTLs explaining phenotypic variance ranging from 10-30% with higher LOD values (3–11). These QTLs will be useful for location specific breeding like *qSPP6-1* (R^2^ = 30%), *qDFF7-1* (R^2^ = 15%), *qSSD8-1* (R^2^ = 22) for New Delhi, Karnal and Aduthurai, respectively. Number of QTLs (57) with minor effect were identified in the present study will be useful based on the fact that quantitative traits were governed by many genes of small effect. These QTLs will help in improvement of yield in smaller way but in cumulative manner.

In the present study, many major QTLs expressing across environments were identified. These QTLs would be highly valuable for breeding the lines with wide adaptation over different locations and crop conditions throughout India. Among robust QTLs expressing over locations, a genomic location at marker interval RM3276-RM5709 on chromosome 4 influencing 4 traits namely flag leaf width (33-53%), spikelets per panicle (9-16%), filled grains per panicle (12-22%) and spikelet setting density (14-19%) and two QTLs i.e., *qPPP4-2* and *qTGW6-1* will be fine mapped to identify candidate genes in future. Most of the major QTLs expressing over locations are flanked by markers at genetic distance of less than 10 cM will be directly be used for marker assisted transfer into the varieties for enhancement of yield through improving the component traits. Pyramiding of the stable QTLs identified in our study with earlier known QTLs of respective traits will be undertaken which will help in raising genetic ceiling to yield.

## Conclusions

The present study confirmed the presence of novel favourable alleles for yield and yield contributing traits among two subspecies i.e., *indica* and *japonica* of cultivated rice (*Oryza sativa* L.) The major robust QTLs are useful for transfer to different genetic backgrounds through marker assisted backcross breeding to break genetic barriers to yield. The novel QTLs identified are highly useful for fine mapping and map based cloning studies. In post genome sequencing era, the availability of complete sequence information and different marker systems for saturation of linkage map will help to understand these QTLs further. Considering the effect and distribution of novel yield influencing QTLs among two cultivated species, further research is needed to unearth and use novel genomic regions influencing yield contributing traits to attain food security.

## Methods

### Plant materials

Pusa1266 is a semi dwarf, late maturing, high tillering, high grain number, high yielding new plant type derivative developed through *indica* X *japonica* hybridization involving Tainan 3, a tropical *japonica* variety, Xiangnuo, an aromatic glutinous *japonica* variety and wide compatibility donors such as N22, Dular and Gharbaran, a high yielding *indica* variety IR72 and number of elite breeding lines that are being developed at Indian Agricultural Research Institute, New Delhi. Pusa1266 was crossed as the female with Jaya, a semi dwarf, high yielding *indica* rice variety. A single F_1_ plant with marker verified hybridity of the cross Pusa1266 × Jaya was selfed to produce F_2_ population and from F_2_ onwards the lines were advanced through single seed descent method [40]. Finally three hundred and ten recombinant inbred lines (RILs) in F_7_ generation were used for QTL mapping.

### Phenotypic evaluation of RILs across locations

The mapping population consisting of 310 F_7_ RILs was evaluated at three locations across India namely, Indian Agricultural Research Institute (IARI), New Delhi , IARI Regional Station, Karnal, Haryana and Rice Breeding and Genetics Research Centre (RBGRC), Aduthurai, Tamilnadu during the wet season, 2006. IARI, New Delhi is located at 28.4°N 77.1°E with an average rainfall of 797.3 mm yr^-1^ with 39 mean rainy days and an average maximum temperature of 34°C in the wet season (June-November). IARI Regional Station, Karnal, Haryana (29.4°N 76.5°E) receives an annual mean rainfall of 1058 mm yr^-1^ with 50 mean rainy days with an average maximum temperature of 32.9°C during wet season. RBGRC, Aduthurai (11°N 79.3°E) located in Tamilnadu, South India, receives a mean rainfall of 1266 mm yr^-1^ in 56 mean rainy days and the mean maximum temperature of 31.8°C during wet season.

For phenotyping across locations, the 310 RILs were transplanted along with the parents of RIL population i.e., Pusa1266 and Jaya, and popular high yielding varieties like Pusa44 (medium duration) and IR64 (mid-early duration) as checks. Each entry was planted with 15 single plants per row at spacing of 30 cm between rows and 20 cm between plants in an augmented randomized complete block design with 5 blocks each with 66 entries including 62 RILs and 4 checks. The randomization procedure was followed as per Federer [41]. Five individual plants were selected from the middle of each row for data collection.

DFF was recorded as the number of days from sowing to 50% of plants with initiation of flowering based on visual observation of the each line. PHT was recorded at the stage of milk development and was measured in cm from base of the plant at soil surface to the panicle tip of main tiller and averaged over five plants. PL was measured in cm from the panicle neck to the tip (excluding awn) at ripening stage, PPP was measured by counting number of panicle bearing tillers per plant at harvesting stage, FLL and FLW of the main tiller was measured in cm at beginning of anthesis, SPP were obtained by counting number of grains (filled and unfilled) per panicle, FGP were obtained by counting number of filled grains per panicle, PSTE was calculated by dividing unfilled spikelets per panicle with total spikelets per panicle expressed as percentage, TGW was measured in grams by weighing 1000 filled grains from each plant , YLD was measured in grams by weighing harvested grains of a plant, SSD was calculated by dividing number of total spikelets by panicle length. The observations for all the traits were averaged over five plants.

### Statistical analysis of the phenotypic data

Mean, standard deviation range of each of the character was obtained using Proc Means of Statistical Analysis System (SAS) v.9.1 [42]. For each location, the data for each character was analyzed as per procedure of augmented randomized complete block designs using Statistical Package for Augmented Designs [43]. The treatment sum of squares was partitioned into sum of squares among RILs, among checks and between RILs and checks. The adjusted means of RILs and checks were obtained and used for further analyses. For making all possible pair wise treatment comparisons, the critical differences were obtained at 5% level of significance for each of the four different kinds of comparisons like between checks, between RILs and check, between RILs of same block as well as between RILs of different blocks [41,44,45]. The homogeneity of error variances over locations were tested using Bartlett’s Chi-square test. When the error variances were found homogeneous, the combined analysis was performed on the original data. If, the error variances were found to be heterogeneous, then the data were transformed by dividing each observation by its corresponding mean square error (Aitken’s transformation) and combined analysis was performed on the transformed data. For performing the combined analysis the model included location, block, genotype and location × genotype effects. Location, block and location × genotype effects were considered as random. The analysis was performed using the SAS code given at Groups of experiments Analysis of Data on Design Resources Server after suitable modifications [46]. The adjusted means of RILs over three locations were subjected to Additive Main effects and Multiplicative Interaction effects (AMMI) analysis. The Pearson’s Product moment correlation coefficients were obtained using PROC CORR of SAS for data combined over all three locations. The significance of correlation coefficients was tested using *t*-test.

### Genomic DNA isolation, polymorphism survey and genotyping of the RIL mapping population

Deoxyribo nucleic acid (DNA) was isolated by Cetyl- trimethyl ammonium bromide (CTAB) method [47] and quantified by using gel electrophoresis in 0.8% agarose gel in 0.5X Tris-acetate-EDTA (TAE) buffer along with known concentrations of λ genomic DNA as standard. The polymerase chain reaction (PCR) was carried out in 96 well PCR plates obtained from Axygen Scientific Inc. Union city CA, USA. The master mix consisted of 25 ng of genomic DNA 0.2 U of *Taq* DNA polymerase, 1 X PCR assay buffer with 1.5 mM MgCl_2_, 12 ng (1.8 picomole) each of forward and reverse primer and 200 μM of dNTP mix in a 10 ul reaction volume. The reaction mix was prepared on ice and the PCR plate was immediately loaded in the thermal cycler (Eppendorf, Biometra or Applied Biosystems USA) for PCR using conditions of (1) initial denaturation at 94°C for 5 min; (2) 35 cycles of 94°C for 1 min, 55-60°C (depending on marker) for 1 min; 72°C for 2 min; (3) final extension at 72°C for 5 min. The PCR products were separated in 3% MetaPhor® agarose gel.

A set of 1063 markers comprising of 521 Rice Microsatellite (RM), 139 Genic Non Coding Microsatellite (GNMS), 388 Hypervariable Simple Sequence Repeat (HvSSR) and 15 gene based markers were used for polymorphism survey between Pusa1266 and Jaya. The primer sequences for RM series markers were obtained from the publications [48-51] and the gramene SSR marker web resource (http://www.gramene.org), GNMS markers [52] and HvSSR markers [53]. A set of 15 gene based primers were designed using the sequences of cloned genes for yield and yield related traits. The genotyping of the RILs was carried out with 162 polymorphic markers, providing genome wide coverage.

### Development of linkage map and QTL mapping

The RIL population was genotyped with 162 polymorphic markers including a gene based marker (*GS3*) and were used to construct a linkage map using the program MAPMAKER [54]. The linkage map covered 2023.1 cM with an average marker interval of 19.64 cM. Most of the marker loci followed expected Mendelian segregation (1:1) for a RIL population. However, segregation distortion was detected for 23 marker loci distributed across 9 chromosomes, with the exception of chromosomes 1, 2 and 11. Of these 23 markers, 7 were skewed towards Pusa1266 and 16 were skewed towards Jaya. Segregation distorted region (SDR) with seventeen markers was found on chromosome 3, 4, 5, 6 and 7 in the same regions where gametophytic or sterility loci (ga/S) have been reported. On chromosome 6, markers with distorted segregation ratio were distributed all along length of chromosome including the ga/S loci. On Chromosome 4, six markers with distorted segregation were in the region where no such loci were reported earlier. The probable reason for segregation distortion might be because of the RIL population involved one NPT parent (Pusa 1266) derived from *indica* X *japonica* cross. The overall level of heterozygosity in the population was calculated to be 0.9%, which was much lower than the expected 1.56% on the basis of six generations. The heterozygotes of individual loci were treated as missing data. The genetic linkage map of RIL population used in the present study was earlier generated by Balram et al. [55]. WinQTLCart version 2.5 [56] was employed for the detection of QTLs. Nomenclature for QTLs was first two or three letter abbreviation followed by the identity of the chromosome on which the QTL is found and a terminal suffix with unique identifier to distinguish multiple QTL on a single chromosome was used [57]. Permutation test was performed with 1000 permutations to identify threshold values of LOD for each trait for declaring a QTL as significant. Simple interval mapping was performed for each location separately. Composite interval mapping was performed by using standard model (model 6) which includes selection of five markers as cofactors (to remove bias that may be caused by QTLs that are linked to the position tested) with window size of 10 cM and tested for QTL positions and effects by forward and backward regression method to identify QTLs with 1 LOD confidence interval. Epistatic QTLs were identified using multiple interval mapping method by scan through QTL mapping file model by setting threshold LOD value of CIM for that trait and 5 cM minimum distance between two QTLs for considering interaction effects and the results pertaining to interacting/epistatic QTLs which explained more than 5% phenotypic variation are reported.

QTL hotspots were identified manually by searching in a sliding window of 20 cM in the original QTL data and the regions with more than three co-locating QTLs in each window region were recorded. The window was advanced in 5 cM steps across the entire genetic map and the maximum number of QTL in a window region was recorded.

QTLs identified in the present study were compared with earlier reports to detect common QTLs across populations. The results from 32 previous rice QTL studies were first examined for data on the 12 traits for comparison. QTLs on the same chromosome as found in the present study were selected for detailed comparisons. Two rice genetic linkage maps, the Cornell RFLP map and the Cornell SSR map, were used to compare QTL locations found in the present study. The QTLs were placed on a framework map based on cross map comparison among the three rice genetic linkage maps. QTLs were identified as potentially novel if the marker intervals harboring QTLs were not significantly overlapping the previously reported marker intervals.

## Abbreviations

NPT, New plant type; RILs, Recombinant inbred lines; QTLs, Quantitative trait loci; AMMI, Additive main effects and multiplicative interaction effects; GXE, Genotype by environment; LOD, Logarithm of odds; DFF, Days to 50% flowering; PHT, Plant height; PL, Panicle length; PPP, Panicles per plant; FLL, Flag leaf length; FLW, Flag leaf width; SPP, Spikelets per panicle; FGP, Filled grains per panicle; PSTE, Per cent sterility; TGW, 1000 grain weight; YLD, Yield per plant; SSD, Spikelet setting density; IARI, Indian agricultural research institute; RBGRC, Rice breeding and genetics research centre; SAS, Statistical analysis system; DNA, Deoxyribonucleic acid; CTAB, Cetyl trimethyl ammonium bromide; TAE, Tris-acetate-EDTA; PCR, Polymerase chain reaction; dNTPs, Deoxyribonucleoticde triphosphate; RM, Rice microsatellites; GNMS, Genic noncoding microsatellites; HvSSR, Hypervariable simple sequence repeats; R2, Phenotypic variance; SDR, Segregation distorted region.

## Competing interests

The authors declare that they have no competing interests.

## Authors' contributions

MBR participated in the design, carried out the field studies and marker studies, analyzed the data and drafted the manuscript. SG associated in the marker studies. SA involved in development of mapping population. TM, participated in the drafting the manuscript. RP participated in design and analysis of phenotypic data. MN, SS associated with field studies. KKV, KVP, NKS participated in the analysis of data and drafting the manuscript. AKS conceived the study, design, coordination, analysis and drafting the manuscript. All authors read and approved the final manuscript.
